# Impact of Combined Neuromuscular Electrical Stimulation (Comb-NMES) on Glucose Signaling and Muscle Myofiber Distribution in a Patient with Acute Spinal Cord Injury and Lower Motor Neuron Lesion

**DOI:** 10.3390/jcm14030876

**Published:** 2025-01-28

**Authors:** Amal Alharbi, Erika Womack, Ceren Yarar-Fisher

**Affiliations:** 1 Department of Physical Therapy, College of Applied Medical Sciences, Qassim University, Buraydah 51452, Saudi Arabia; 2 Department of Biochemistry, Molecular Biology, Entomology, and Plant Pathology, Mississippi State University, Starkville, MS 39762, USA; ewomack@mscl.msstate.edu; 3 Department of Physical Medicine and Rehabilitation, Ohio State University, Columbus, OH 43210, USA; 4 Department of Neuroscience, Ohio State University, Columbus, OH 43210, USA

**Keywords:** spinal cord injury, lower motor neuron lesion, combined neuromuscular electrical stimulation, muscle glucose uptake, muscle atrophy

## Abstract

**Introduction:** This case report examines the impact of a novel combined neuromuscular electrical stimulation (Comb-NMES) regimen on muscle glucose signaling, fiber type distribution, and metabolic function in a patient with acute spinal cord injury (SCI) and lower motor neuron lesions (LMNLs). **Case Report:** A 32-year-old male with complete T9 SCI underwent a ten-session Comb-NMES intervention targeting the quadriceps. Muscle biopsies and blood samples were analyzed pre- and post-intervention to evaluate changes in muscle fiber types, key metabolic proteins, fasting insulin, glucose, and lipid profiles. **Results:** The intervention led to a 74.7% and 28.2% reduction in fasting insulin and glucose, respectively. Muscle analysis showed significant increases in CaMK II, Hexokinase II, and IRS-1, indicating improved glucose metabolism. **Conclusions:** Comb-NMES training markedly improved metabolic control and muscle glucose metabolism in a patient with acute SCI and LMNLs. Enhanced insulin sensitivity and glucose utilization were evidenced by upregulated metabolic proteins, which suggests that Comb-NMES is a promising intervention for improving muscle and metabolic health in SCI patients with LMNLs. Further studies are needed to confirm these benefits and explore the long-term effects.

## 1. Introduction

Spinal cord injury (SCI) is often a devastating traumatic event that leads to serious secondary health complications, including increased risks of diabetes and cardiovascular diseases [1,2]. Following SCI, significant muscle atrophy typically develops below the lesion site, primarily due to prolonged diminished contractile activity [3]. This muscle atrophy is typically marked by a decrease in muscle mass and cross-sectional area, particularly affecting muscles innervated by the spinal cord segments below the injury level, resulting in pronounced weakness and diminished function [4]. Additionally, these below-injury-level muscles display a higher proportion of fast glycolytic fibers, which, despite their capacity for quick and intense contractions, are implicated in reduced endurance and suboptimal muscle performance [5]. However, when SCI affects all the lower motor neurons (LMNs), the atrophy of skeletal muscle groups becomes particularly severe [6].

Approximately 20–25% of individuals with SCI experience LMNLs, resulting in profound muscle atrophy due to denervation, with associated adipose infiltration and fibrosis, which further impair muscle function and metabolic health. These changes place individuals with LMNLs at heightened risk of metabolic and cardiovascular complications compared to those with upper motor neuron (UMN) lesions [7]. UMN lesions and LMN lesions exhibit distinct pathophysiological profiles. UMN lesions are characterized by spasticity, hyperreflexia, and disuse atrophy, primarily the disruption of descending motor pathways. In contrast, LMN lesions result in denervation atrophy, which is marked by flaccidity, loss of reflexes, and significant muscle wasting [8]. When both UMN and LMN lesions coexist, as seen in injuries involving thoracic segments (e.g., T9) and associated lumbar segments (e.g., L2–L4), the combined impact poses unique challenges [8]. LMN lesions, in particular, disrupt the final common motor pathway, leading to denervation and loss of neural circuits necessary for effective muscle stimulation [9]. Doherty et al. emphasize that distinguishing between UMN and LMN involvement is critical for managing SCI patients and optimizing therapeutic interventions [8]. Muscle atrophy caused by upper motor neuron lesions remains consistent from three to twenty years post-SCI [10]. This situation contrasts starkly with SCIs that affect both upper and lower motor neurons (LMNs), as such injuries result in the actual denervation of the muscles. The condition is especially dire when a complete transverse SCI affects all LMNs of the involved muscles. These completely denervated muscles quickly become incapable of maintaining tension during tetanic contractions induced by electrical stimulation [11]. Eventually, they lose excitability with conventional electrical stimulators, leading to severe, enduring atrophy as muscle fibers are replaced by adipocytes and collagen [12,13]. Therefore, there has been significant interest in the use of electrical stimulation to restore/maintain the structure and function of skeletal muscles in SCI individuals [14,15,16]. The current research literature does not adequately address the effects of neuromuscular electrical stimulation (NMES) on muscle health and metabolic functions in individuals with acute SCI who also have LMNLs.

In this case report, we report on the efficacy of a novel Combined NMES (Comb-NMES) regimen on the distribution of muscle fiber types and the signaling pathways involved in muscle glucose uptake. The protocol was designed to be manageable for individuals to perform on their own, whether seated in wheelchairs or lying in hospital beds. Therefore, the goal of this report is to document the effect of the Comb-NMES on muscle health and metabolic function in acute SCI individuals with LMN lesions that exhibited markedly reduced muscle contractions with fibrillations in addition to a pronounced reduction in fasting insulin levels and glucose concentrations.

## 2. Case Presentation

A 32-year-old man had suffered a complete SCI (T9, AISA A) secondary to a gunshot wound. The patient stayed at the Traumatic Intensive Care Unit in UAB Hospital (Birmingham, UK) for two weeks. Then, he was transferred to the Spain Rehabilitation Center (SRC) to receive medical care after the traumatic injury. He remained in the intervention for 23 days with a total of 10 sessions given three times/week. He had no previous history of cardiovascular or metabolic disorders. The patient enrolled in this study after signing a consent form. The study protocol was approved by the University of Alabama in Birmingham’s (UAB) Institutional Review Board. This case report is part of a large study that investigated the effect of combined neuromuscular electrical stimulation on muscle health and metabolism after acute SCI. This study was registered at clinicaltrials.gov (NCT03204240).

The Comb-NMES regimen we have designed incorporates dynamic contractions through electrical stimulation at a high frequency (50 Hz trains with 450 µs biphasic pulses) for resistance training, which aimed to induce a tetanic muscle contraction, facilitating complete knee extension (concentrical and eccentrically) with twitch contractions elicited by low-frequency electrical stimulation (5 Hz with a pulse duration/interval of 200/50 µs) for aerobic contraction, targeting the quadriceps muscle group of both legs. This twitch contraction contracts the muscle isometrically. Two electrodes (typically 7.6 × 12.7 cm) were used for stimulation, placed on the proximal region of the vastus lateralis and the distal part of the vastus medialis.

In the course of the intervention with Comb-NMES, it was observed that the patient exhibited markedly reduced muscular contractions, which were palpable but not visually discernible in the lower extremities. Fibrillation, decreased reflexes, and tone were observed during the session. While standard diagnostic measures such as electromyography (EMG) to confirm LMN lesions were not employed, these clinical findings raise the possibility of an underlying LMN injury. It is important to note that the thoracic vertebrae are defined by their corresponding vertebral levels; however, spinal cord segments do not always align anatomically with the vertebrae. For instance, the lumbar spinal cord is situated between the T9 and T11 vertebrae. Therefore, a gunshot wound at the T9 level can result in damage to the lumbar spinal cord segments L2–L4 [17]. It is proposed that in the SCI population, 20–25% suffer from LMN lesions [7]. This speculation is grounded in the understanding that LMN lesions can result in significant deficits in voluntary and reflexive muscle contractions, which aligns with the diminished responsiveness observed in the patient’s muscles to the electrical stimulation [7]. However, it is important to note that this interpretation is put forward with caution and acknowledges the limitation of not having utilized standard tests that could offer a definitive diagnosis of LMN damage.

The patient received the Comb-NMES training protocol, which is published elsewhere [18]. Briefly, training was performed while the patient was seated in a wheelchair or bed with their knee flexed between 70 and 90 degrees. The training was conducted three times weekly under professional supervision using the TheraTouch 4.7 (Rich-Mar, Inola, OK, USA) stimulation device with self-adhesive 7.6 × 13 cm electrodes (Axelgaard ValuTrode, Fallbrook, CA, USA) for quadriceps stimulation. The Comb-NMES intervention combined resistance and aerobic exercises. Resistance exercises involved four sets of 10 quadricep contractions using 50 Hz, 450 µs pulses, with intensity modulated from 0 to 200 mA to achieve full knee extension, and ankle weights were adjusted as needed. Aerobic exercises started with 10 min of 2 Hz twitch stimulation and increased to 30 min at 6 Hz, with intensity set at 175 mA.

## 3. Clinical and Laboratory Procedures

Muscle samples were obtained from the vastus lateralis using a Bergstrom-type needle under local anesthesia (1% lidocaine). For immunohistochemistry, small muscle tissue samples (50–70 mg) were mounted cross-sectionally and quick-frozen in nitrogen-cooled isopentane, with the remaining tissues snap-frozen for subsequent biochemical analyses.

**Figure 1:**
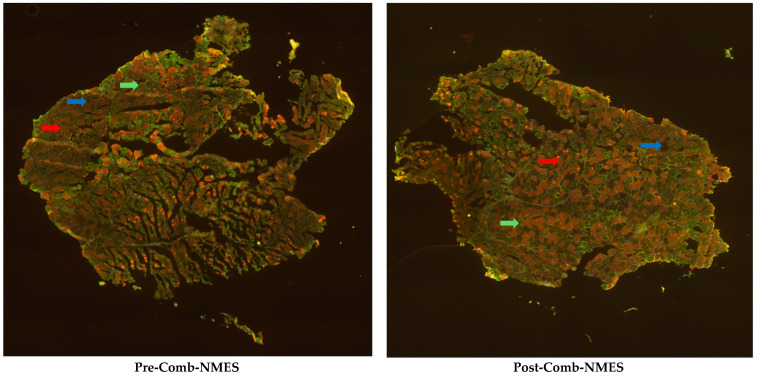
Fluorescent immunohistochemistry images from the vastus lateralis muscle of the patient. The red arrow indicates type I, the green arrow indicates type IIa, and the blue arrow indicates type IIx muscle fibers.

Frozen muscle samples were sectioned into 6 µm using a cryostat. Myofiber types I, IIa, and IIax/IIx were identified through immunohistochemistry. Sections were stained with primary antibodies (NCL-MHCs for MHC I, NCL-MHCf for MHC II, and anti-laminin) and secondary antibodies (ALEXA Fluor 594 and 488) following the standard protocols. Hybrid IIax fibers were combined with type IIx fibers due to their prevalence (Figure 1).

Muscle protein lysates were prepared, homogenized with protease and phosphatase inhibitors, and centrifuged. The protein content was determined using the bicinchoninic acid method. Proteins, including glucose transporter 4 (GLUT 4), total and phosphorylated AMP-activated protein kinase-α (AMPK-α), calcium/calmodulin-stimulated protein kinase II (CaMKII), Akt substrate of 160 kDa (AS160), Akt, Hexokinase II, glycogen synthase (GS), and insulin receptor substrate 1 (IRS-1), were separated via SDS-PAGE and transferred to nitrocellulose membranes. Membranes were blocked, probed with specific antibodies, and analyzed using densitometry. The myofiber type distribution of VL was determined for the total number of 925 fibers (type I: 394, type IIa: 521, type IIx: 10) in the pre-training and for 873 fibers (type I: 406, type IIa: 443, type IIx: 24) in the post-training.

### Blood Glucose, Insulin, and Lipid Measurements

Serum glucose, cholesterol, low-density lipoprotein (LDL), high-density lipoprotein (HDL), and triglycerides were measured using automated analyzers, with LDL calculated via the Friedewald method. Serum insulin levels were determined by immunofluorescence. This methodological approach is adapted from previously published work (Alharbi et al., 2023 [18]).

## 4. Statistical Analysis

Due to the case report nature of this study, conventional statistical methods were not applicable. Descriptive analysis was used to detail changes in the patient’s measurements pre- and post-Comb-NMES intervention, with percent change calculations and visual representations through protein blots and microscopic imaging.

## 5. Results

### 5.1. Fasting Insulin and Glucose Levels

A significant reduction (74.7%) was observed in the fasting insulin levels (pre: 49.50 µIU/mL, post: 12.50 µIU/mL), and a moderate decrease (28.2%) in fasting glucose levels was observed (pre: 117.00 mg/dL, post: 84.00 mg/dL).

### 5.2. Fasting Lipid Profile

There were slight changes in the lipid profiles. For example, the fasting triglyceride levels increased by 8% (pre: 125.0 mg/dL, post: 135.0 mg/dL). The LDL and HDL levels declined by 1.6% and 8.9%, respectively (pre LDL: 191.0 mg/dL, post LDL:188.0 mg/dL; pre HDL: 45 mg/dL, post HDL: 41 mg/dL). The total cholesterol levels showed a slight increase (2.8%; pre: 247 mg/dL, post: 254 mg/dL).

### 5.3. Skeletal Muscle Intracellular Signaling

**Figure 2:**
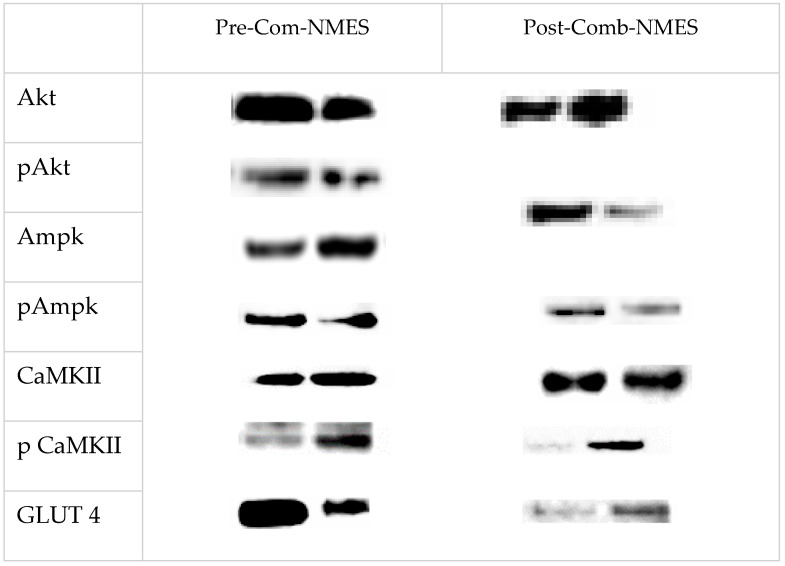
Immunoblots of studied proteins. protein kinase B, Akt; phosphorylated protein kinase B, pAkt; AMP-activated protein kinase, AMPK; phosphorylated AMP-activated protein kinase, pAmpk; Ca^2+^/calmodulin-dependent protein kinase, (CaMK) II; phosphorylated Ca^2+^/calmodulin-dependent protein kinase, (CaMK) II; glucose transporter 4, GLUT−4.

The AKT and pAKT_Ser473 levels decreased (55% and 17%, respectively). In addition, AMPK and pAMPK_Thr172 slightly decreased (9% and 2.6%, respectively). The CaMKII and pCaMKII_Thr286 levels significantly increased (76%, and 159%, respectively). The GLUT 4 expression was reduced by 46.7%. Glycogen synthase increased by 42%, and phosphorylated GS_Ser641 markedly decreased by 72%. AS160 increased by 0.4% and pAS160_Ser318 decreased by 29%. Remarkably, Hexokinase II increased by 451% and IRS1 increased by 85% post-Comb-NMES (Figure 2).

### 5.4. Myofiber-Type Distribution

There was a slight increase in MHC I fibers (3%) and a drastic increase in MHC IIx fibers (140%). Conversely, MHC IIa fibers slightly decreased (15%).

## 6. Discussion

A pronounced reduction in fasting insulin levels (pre: 49.5 µIU/mL, post: 12.5 µIU/mL) and glucose concentrations (pre: 117.0 mg/dL, post: 84.0 mg/dL) indicates a meaningful improvement in metabolic control following Comb-NMES training. Insulin, a hormone responsible for controlling blood glucose levels, plays a crucial role in metabolic health. Lower fasting insulin levels may reflect increased insulin sensitivity, while reduced fasting glucose levels can denote better glucose utilization [19]. Both measures are essential indicators of improved metabolic function. These findings suggest that Comb-NMES enhances glucose utilization, potentially through increased muscular uptake and utilization, possibly due to enhanced insulin sensitivity. The overall lack of change in the lipid profiles indicates that Comb-NMES was not effective in altering circulating lipid levels. The lack of significant changes may be attributed to the short duration of the intervention. Further research with longer intervention periods is necessary to better understand the potential effects of Comb-NMES on lipid metabolism.

The Comb-NMES intervention influenced several key proteins involved in muscle glucose uptake and metabolism. The observed substantial increases in CaMKII and p CaMKII _Thr286 levels (76% and 159%, respectively) highlight the activation of this pathway via Comb-NMES. CaMKs are critical regulators of muscle metabolism, responding to changes in intracellular calcium (Ca^2+^) levels. The increase in Ca^2+^ levels is a fundamental aspect of muscle contraction [20]. CaMKs can regulate glucose uptake independently of AMPK signaling [20]. The marked increase in CaMKII phosphorylation suggests that Comb-NMES effectively stimulates this signaling pathway in the denervated muscle. In addition, this enhanced response could be attributed to the altered muscle fiber composition in SCI, which tends to have a higher proportion of type II fibers that are more responsive to Ca^2+^-mediated signaling [21,22].

IRS-1’s robust response can be attributed to its pivotal role in mediating the effects of insulin on muscle cells. During and after exercise, there is an increase in the phosphorylation of IRS-1, which enhances its activity, thereby amplifying downstream signaling that promotes glucose uptake and glycogen synthesis [23]. Electrical muscle stimulation can mimic the effects of physical exercise; therefore, Comb-NMES could lead to increased insulin receptor activation and the subsequent upregulation of IRS-1. In addition, both acute and chronic exercise can enhance the expression and activation of insulin signaling molecules to improve insulin sensitivity [23]. As a result, the upregulating response of IRS-1 likely reflects an adaptive mechanism to optimize glucose uptake and utilization in response to increased metabolic demand in the denervated muscle.

Hexokinase II and GS both play critical roles in glucose metabolism. Hexokinase II is responsible for phosphorylating glucose to glucose-6-phosphate, a necessary step for its utilization and storage [22]. Its 451% increase suggests a heightened capacity for glucose phosphorylation, possibly as a response to increased glucose uptake stimulated by Comb-NMES. Glycogen synthase, which showed a 42% increase, is crucial for glycogen synthesis. This aligns with exercise-induced adaptations where the muscle stores more glycogen to prepare for future energy demands. The increase in both enzymes suggests an enhanced capacity for glucose utilization and storage, supporting the muscle’s energetic needs during and after exercise [24].

GLUT 4 is crucial for glucose uptake in the skeletal muscle and is typically upregulated in response to exercise [20]. However, in this study, we did not observe an increase in the GLUT 4 levels. This might be due to the specific type of electrical stimulation used (Comb-NMES) and the short nature of the training.

One of the most common changes in muscles after SCI is the transformation of muscle fibers from slow-twitch to fast-twitch fibers [25]. Slow-twitch fibers, also known as type I fibers, are adapted for endurance and sustained activity, using aerobic metabolism to efficiently produce energy with a high fatigue resistance. In contrast, fast-twitch fibers, or type II fibers, are designed for quick bursts of speed and power, relying more on anaerobic metabolism [26]. Our findings showed a substantial increase in type IIx fibers (140%) and a minimal increase in type I fibers (3%), while type IIa fibers slightly decreased (15%) (Figure 1). Previous work demonstrated that NMES resistance training successfully shifted type IIx to type IIa in chronic SCI individuals [16]. Therefore, we assume that the increase in type IIx and decrease in type IIa in our study may be related to the NMES resistance training duration being inadequate for the denervated muscle to enhance such a transition, as seen in other studies. Additionally, NMES aerobic training has been shown to increase the distribution of type I fibers in chronic SCI [27]. In our current case, type I fibers did not change significantly; therefore, we speculate that the training intensity and/or duration was insufficient to increase type I fiber distribution in a denervated muscle.

There remains a challenge in determining whether the observed metabolic improvements are primarily attributable to direct muscle effects or to systemic adaptations, including pancreatic insulin secretion. While this study focused on the effects of NMES on skeletal muscle metabolism, we acknowledge that systemic factors, such as enhanced insulin secretion or altered hormonal regulation, may have also contributed to the observed outcomes. The improvements in glucose metabolism could reflect a complex interplay between localized muscle adaptations and systemic metabolic responses. Future studies should incorporate methodologies to differentiate these effects, such as measuring changes in pancreatic function alongside detailed muscle biopsies. This approach would provide a more comprehensive understanding of the mechanisms underlying NMES-induced metabolic benefits. Additionally, we acknowledge the limitations of analyzing only the vastus lateralis muscle in this case report. While the scope of this study was limited to the lower extremity, future research comparing muscle changes in the upper and lower limbs is necessary, especially in individuals with injuries above the T9 level. Including upper limb muscle biopsies would provide a more comprehensive understanding of systemic muscle responses to NMES interventions. This would enhance our ability to assess the generalizability of the findings and to understand the distinct and combined responses of the upper and lower limb muscles to NMES. Future studies should incorporate follow-up assessments to evaluate the durability of the effects post-intervention and explore potential strategies to sustain the benefits of NMES in the long term.

## 7. Conclusions

The current case report demonstrates that Comb-NMES training can improve fasting glucose and insulin levels in a patient with acute SCI and LMNLs, indicating enhanced metabolic control. These improvements are likely driven by the upregulation of key proteins involved in muscle glucose uptake signaling pathways. While the Comb-NMES intervention did not significantly alter lipid profiles or increase type I muscle fiber distribution, future research with extended intervention periods is warranted to fully elucidate the long-term effects of Comb-NMES.

## Data Availability

The data that support the findings of this study are available on request from the corresponding author [CYF].
